# Thyroid dysfunction in pediatric Fontan patients is associated with unfavorable hemodynamic status and severity of protein-losing enteropathy: A report from the Fontan care network

**DOI:** 10.1016/j.ijcchd.2023.100475

**Published:** 2023-09-12

**Authors:** Joszi Sweer, Ingo Germund, Markus Khalil, Christian Apitz, Kim ten Dam, Stefanie Wendt, Narayanswami Sreeram, Floris E.A. Udink ten Cate

**Affiliations:** a Department of Pediatric Cardiology, Academic Center for Congenital Heart Disease, Amalia Children's Hospital, Radboud University Medical Center, Nijmegen, the Netherlands; b Department of Pediatric Cardiology, Heart Center Cologne, University Hospital Cologne, Cologne, Germany; c Pediatric Heart Center, Justus-Liebig University, Giessen, Germany; d Department of Pediatric Cardiology, University Children's Hospital Ulm, Ulm, Germany; e Department of Cardiac Surgery, Heart Center Cologne, University Hospital Cologne, Cologne, Germany

**Keywords:** Fontan, Protein-losing enteropathy, Thyroid function, Heart failure

## Abstract

**Background:**

Thyroid dysfunction may have adverse effects on Fontan hemodynamics. Data on thyroid function in pediatric Fontan patients with or without protein-losing enteropathy (PLE) are limited.

**Methods:**

This retrospective multicenter study included 67 Fontan patients (median age 10.9 years; 35.8% female; 28.4% PLE) in whom thyroid function testing was performed.

**Results:**

Subclinical hypothyroidism (SHT) was present in 16 (23.9%) patients. Subjects with SHT had significantly lower systolic blood pressure (p = 0.014) and body weight z-score (p = 0.006), were in a worse New York Heart Association (NYHA) functional class (p = 0.004), were more often pacing dependent (p = 0.007), and were more likely to have PLE (p = 0.033, 8/19 (42.1%) patients). Serum thyroid stimulating hormone (TSH) levels were significantly higher in patients with NYHA class ≥ II (p = 0.005), significant atrioventricular valve regurgitation (p = 0.023), elevated serum natriuretic peptides (p = 0.031), and in those with PLE (p = 0.002). Patients with active PLE had significantly higher TSH levels than those in remission (p = 0.003). A strong inverse relationship was found between lower free triiodothyronine (fT3) levels and natriuretic peptides (r: −0.599, p = 0.040). Using binary logistic regression analysis we found that worse NYHA class was an independent predictor of SHT (OR 4.2; 95% CI 1.1–16.1, p = 0.036).

**Conclusions:**

Subclinical thyroid dysfunction is common in Fontan, particularly in patients with hemodynamic derangements and PLE. Future studies are needed to address the prognostic implications of thyroid dysfunction in the Fontan population.

## Highlights

• Alterations in thyroid-axis function is common in Fontan patients, and includes a wide spectrum of abnormalities, such as subclinical hypothyroidism (SHT), higher thyroid stimulating hormone (TSH) levels, and lower free triiodothyronine (fT3) serum levels.
• Higher New York Heart Association functional class (NYHA ≥ II) was an independent risk factor of SHT.
• Subclinical thyroid dysfunction was found in 42% of Fontan patients with PLE, particularly in those with active disease.
• Patients with significant valve regurgitation, RV dominance, and elevated natriuretic peptide levels were more likely to have thyroid abnormalities, suggesting an association between thyroid-axis and Fontan hemodynamics.
• Future studies are needed to address the clinical and prognostic implications of subclinical thyroid dysfunction in the Fontan population.

## 1 Introduction

Thyroid function abnormalities are common in patients with congenital heart disease and may impair cardiovascular function and outcome [[1], [2], [3]]. Even subclinical hypothyroidism (SHT), a clinical state of elevated thyroid stimulating hormone (TSH) with normal serum thyroxine (T4) levels, converts additional cardiovascular risk, especially in those with chronic heart failure [[4], [5], [6], [7]]. Many Fontan patients experience progressive hemodynamic derangements over time [8]. Thyroid hormones have a complex interplay with the cardiovascular system through multiple physiological mechanisms [3,4]. For example, triiodothyronine (T3) molecules, produced in peripheral tissues through enzymatic conversion of T4 to T3, play an essential role in maintaining optimal myocardial relaxation and normal peripheral vascular resistance, both important features of a good Fontan circulation [4,[8], [9], [10]]. Thyroid abnormalities may therefore contribute to the progression of end-organ dysfunction in Fontan patients. However, data on thyroid function status in pediatric Fontan patients are limited, and potential risk factors or associations for thyroid dysfunction have not been fully elucidated [2,5,7]. Furthermore, the prevalence and severity of thyroid dysfunction has not been studied in Fontan patients with protein-losing enteropathy (PLE), a devastating disease associated with multiple co-morbidities and poor outcome [11]. We hypothesize that thyroid dysfunction in PLE may be secondary to enteric protein-loss, or may reflect the severity of the illness, as chronic disease per se is frequently associated with thyroid hormone alterations in the absence of thyroid pathology [3,4].

Therefore, the objectives of this study were to (1) assess the prevalence of thyroid dysfunction in a multicenter cohort of Fontan patients with or without PLE who had thyroid function testing as part of routine screening and (2) identify risk factors associated with thyroid abnormalities in these patients.

## 2 Methods

### 2.1 Study design and definitions

A retrospective chart review was performed of all pediatric Fontan patients (age ≤18 years) routinely followed at one of the participating institutions of the Fontan Care Network (Heart Center Cologne, Radboud University Medical Center, Children's Hospital Ulm, Pediatric Heart Center Giessen) in whom the thyroid function was measured between January 2013 and December 2019. Exclusion criteria were Down syndrome, previous thyroid disease, and treatment with thyroid altering drugs (amiodarone, levothyroxine, or thiamazol). The local institutional review boards of all participating centers approved this study. This study was performed according to the local code of research and data protection rules.

Medical records were searched for anthropometric and clinical data, cardiac anatomy, New York Heart Association (NYHA) functional class, and history of PLE. Gender-specific z-scores were calculated for height, weight, and body mass index (kg/m^2^) relative to age using national pediatric reference values. Echocardiographic studies were reviewed. During the echocardiographic investigations of Fontan patients at the participating institutions, subcostal, apical, long-axis and short-axis images are routinely obtained. Evaluation of ventricular function is predominantly based on visual assessment of these images. This is part of the routine echocardiographic protocols. The principle investigator at each center, all experienced senior pediatric cardiologists, was asked to visually asses global ventricular function using these images. Global ventricular systolic function was then qualitatively assessed and classified as normal/mild impaired, or moderately/severely impaired, as described previously [[12], [13], [14]]. Severity of atrioventricular valve (AVV) regurgitation was graded as none/mild or moderate/severe, as described previously [[12], [13], [14]]. Significant AVV regurgitation was defined as moderate or severe AVV regurgitation on echocardiography. A diagnosis of PLE was made according to recent recommendations [15]. In addition, PLE remission was defined as normal serum albumin levels without clinical symptoms at the time of thyroid testing; patients not in remission were classified as having active disease [16].

TSH, free T4 (fT4), and free T3 (fT3) measurements were collected. Subclinical hypothyroidism was defined as TSH levels above 4.5 mU/L with free T4 levels within the normal range [17,18]. We also calculated the ratio between fT3 and fT4 serum levels (fT3/fT4 ratio), a marker of decreased serum T3 levels and low T3 syndrome [19,20]. Reduced serum T3 levels have been associated with adverse events and all-cause mortality in patients with cardiovascular disease [4,9,19]. Data on complete blood count, liver function, serum electrolytes, protein levels, N-terminal pro brain natriuretic peptide (NTproBNP) or brain natriuretic peptide (BNP), and renal function were collected. Using the white blood cell count, we calculated the neutrophil-to-lymphocyte ratio (NLR), and platelet-to-lymphocyte ratio (PLR), which are surrogate markers of systemic inflammation [[21], [22], [23]]. NTproBNP and BNP levels were considered elevated if levels were above the 97.5th percentile for the subjects specific age group [[24], [25], [26]]. The glomerular filtration rate (eGFR) was estimated from the serum creatinine level and height according to the Schwartz formula [27,28]. Renal dysfunction was defined as a eGFR of <90 ml/min/1.73 m^2^ [27,28].

### 2.2 Statistical analysis

Data are presented as frequency (percentage) for categorical variables and mean ± standard deviation (SD) or median (interquartile range) for continuous variables. Normality of measurements were tested using the Shapiro-Wilk test. Patient and clinical characteristics of patients with and without thyroid dysfunction were compared using Fisher's exact test for categorical variables and either Student's t-test or Wilcoxon Rank Sum test for continuous variables, as appropriate. Spearman's rank correlation coefficient was used to evaluate associations between thyroid function and clinical and laboratory measures. Binary logistic regression was used to assess the relationship between SHT and covariates. A p value < 0.05 was considered statistically significant. Statistical analyses were performed with SPSS Version 25 (Chicago, IL, USA).

## 3 Results

Sixty-seven Fontan patients (median age 10.9 years; 35.8% female) in whom thyroid function was tested were included in this study. None of the included patients received amiodarone, levothyroxine, or thiamazol. The clinical and demographic characteristics of the study group are summarized in Table 1. Hypoplastic left heart syndrome (HLHS) was the most common cardiac diagnosis (50.7%), and PLE was diagnosed in 19 patients (28.4%). Moderate or severe AVV regurgitation was found in 18 patients (26.9%). Patients with significant AVV regurgitation were more likely to be in NYHA class ≥ II (11/22 (50.0%) vs 7/45 (15.6%), p = 0.007) or have elevated serum NTproBNP/BNP levels (11/13 (84.6%) vs 6/47 (12.8%), p < 0.001). Renal dysfunction (eGFR <90 ml/min/1.73 m^2^) was seen in 16/66 patients (24.2%), and was more common in patients with a morphological systemic right ventricle (SRV: 14/27 patients (51.9%) vs non-SRV: 2/25 (8.0%); p = 0.019). There was only 1 patient with a eGFR <60 ml/min/1.73 m^2^.

**Table 1:** Demographic and clinical characteristics of the study cohort.

variable	All patients	Subclinical hypothyroidism		P-value
		yes	no	
Clinical features				
n	67	16 (23.9%)	51 (76.1%)	
Age (years)	10.9 (6.5–15)	12.5 (8.5–16.2)	10.4 (6.1–13.9)	0.100
Age at time of Fontan (years)	2.5 (2.2–3.4)	2.4 (2.2–2.7)	2.7 (2.3–3.5)	0.565
Female sex	24 (35.8%)	6 (37.5%)	18 (35.3%)	0.549
Systolic BP (mmHg)	110 ± 12	103 ± 11	112 ± 12	**0.014**
Diastolic BP (mmHg)	62 ± 11	58 ± 7	63 ± 11	0.097
Systemic oxygen saturation (%)	96 (94–97)	95 (91–96)	96 (94–98)	0.082
Weight	36 ± 19	35 ± 16	37 ± 20	0.687
Weight z-score	−0.5 ± 1.7	−1.5 ± 1.9	−0.2 ± 1.5	**0.006**
Height	136 ± 23	136 ± 19	136 ± 24	0.943
Height z-score	−1.2 ± 1.8	−2.1 ± 2.4	−0.8 ± 1.5	0.061
BSA	1.2 ± 0.4	1.1 ± 0.3	1.1 ± 0.4	0.765
BMI	18.5 ± 4.3	17.8 ± 3.7	18.7 ± 4.4	0.514
BMI z-score	0.1 ± 1.4	−0.4 ± 1.6	0.3 ± 1.2	0.084
NYHA Class				**0.004**
Class I	45 (67.2%)	6 (37.5%)	39 (76.5%)	
Class II or higher	22 (32.8%)	10 (62.5%)	12 (23.5%)	
Ventricular dysfunction				0.556
No or mild	57 (85.1%)	14 (87.5%)	43 (84.3%)	
Moderate or severe	10 (14.9%)	2 (12.5%)	8 (15.7%)	
AV valve regurgitation				0.216
No or mild	49 (73.1%)	10 (62.5%)	39 (76.5%)	
Moderate or severe	18 (26.9%)	6 (37.5%)	12 (23.5%)	
Cardiac diagnosis				0.259
HLHS	34 (50.7%)	11 (68.8%)	23 (45.1%)	
TA	7 (10.4%)	1 (6.3%)	6 (11.8%)	
DILV	2 (3.0%)	0 (0%)	2 (3.9%)	
DORV	3 (4.5%)	2 (12.5%)	1 (2.0%)	
PA	6 (9.0%)	0 (0%)	6 (11.8%)	
Unbalanced AVSD	2 (3.0%)	0 (0%)	2 (3.9%)	
Single ventricle	13 (19.4%)	2 (12.5%)	11 (21.6%)	
Type of ventricle				0.568
Right dominant systemic ventricle	42 (62.7%)	12 (75.0%)	30 (58.8%)	
Left dominant systemic ventricle	21 (31.3%)	4 (25.0%)	17 (33.3%)	
Indeterminant ventricle	4 (6.0%)	0 (0%)	4 (7.8%)	
Heterotaxy	3 (4.5%)	0 (0%)	3 (5.9%)	
Type of Fontan				0.508
Lateral tunnel	3 (4.5%)	0 (0%)	3 (5.9%)	
Extracardiac conduit	62 (92.5%)	15 (93.8%)	47 (92.2%)	
Other	2 (3.0%)	1 (6.3%)	1 (2.0%)	
Pacemaker	7 (10.4%)	5 (31.3%)	2 (3.9%)	**0.007**
PLE	19 (28.4%)	8 (50.0%)	11 (21.6%)	**0.033**
Medication				
Coumarin	37 (55.2%)	11 (68.8%)	26 (51%)	0.204
Acetylsalicylic acid	23 (34.3%)	4 (25%)	19 (37.3%)	0.368
ACE inhibitor	31 (46.3%)	8 (50%)	23 (45.1%)	0.732
Sildenafil	14 (20.9%)	4 (25%)	10 (19.1%)	0.198
Diuretics	12 (17.9%)	8 (50%)	4 (7.8%)	**< 0.001**
Corticosteroids	8 (11.9%)	4 (25%)	4 (7.8%)	**0.029**
Aldosterone antagonist	22 (32.8%)	10 (62.5%)	12 (23.5%)	**0.006**
β-blocker	15 (22.4%)	6 (37.5%)	9 (17.6%)	0.161

Data are presented as n (%) for categorical variables and median [25th – 75th interquartile range] or mean ± standard deviation for continuous variables. AVSD = atrioventricular septal defect; BMI = body mass index; BP = blood pressure; BSA = body surface area; DILV = double inlet left ventricle; DORV = double outlet right ventricle; HLHS = hypoplastic left heart syndrome; NYHA = New York Heart Association classification; PA = pulmonary atresia; PLE = protein-losing enteropathy; TA = tricuspid atresia.

Sixteen Fontan patients (23.9%) were diagnosed with SHT. There were no patients with clinically overt hypo- or hyperthyroidism. Subjects with SHT had a significantly lower systolic blood pressure (BP) (p = 0.014) and body weight z-score (p = 0.006), were in a worse NYHA class (p = 0.004), and were more often pacing dependent (p = 0.007). Furthermore, patients with PLE were also more likely to have SHT (p = 0.033, 8/19 (42.1%) of patients) in comparison to those without PLE.

Laboratory measures are summarized in Table 2. Serum TSH levels were significantly higher in subjects with SHT (p < 0.0001). IgG levels were significantly lower in Fontan patients with SHT (p = 0.038). In addition, median TSH levels were significantly higher in Fontan patients with a NYHA class ≥ II (p = 0.005), moderate or severe AVV regurgitation (p = 0.023), elevated serum levels of NTproBNP or BNP (p = 0.031), and in those with PLE (p = 0.002, Table 3). Interestingly, Fontan patients with active PLE had significantly higher TSH levels than PLE patients who were in remission (median TSH levels: 4.9 (3.9–5.8) mU/L vs 3.3 (2.9–4.3) mU/L, p = 0.003). Significantly increased TSH levels were not found in patients with ventricular dysfunction (p = 0.972), a SRV (p = 0.100) or in those with renal dysfunction (p = 0.435).

**Table 2:** Laboratory measures.

Variable	Number of patients with each test	All patients	Subclinical hypothyroidism		P-value
			yes	no	
Laboratory parameters					
Hemoglobin (g/dL)	67	13.9 (12.7–14.7)	14.1 (13.6–15.6)	13.4 (12.7–14.5)	0.050
MCV (fl)	67	81 (78–85)	83 (79–86)	82 (78–85)	0.696
WBC count (x10^9^/L)	67	7.0 (5.1–8.6)	7.1 (5.9–9.0)	6.3 (4.9–8.4)	0.378
Platelet (x10^9^/L)	67	253 (193–306)	244 (194–336)	237 (184–286)	0.503
% Neutrophils	52	57 (51–64)	61 (58–70)	56 (50–64)	0.183
Neutrophils (x10^9^/L)	52	4.3 (2.9–5.6)	4.5 (3.3–6.0)	3.6 (2.9–5.2)	0.246
% Lymphocytes	47	26 (21–32)	26 (7–30)	28 (21–32)	0.365
Lymphocytes (x10^9^/L)	51	1.8 (1.3–2.4)	1.6 (0.7–2.4)	1.6 (1.3–2.4)	0.632
Neutrophil-lymphocyte ratio	50	2.9 (1.6–3.1)	2.3 (1.9–5.9)	2.1 (1.6–3.1)	0.368
Platelet-lymphocyte ratio	51	175 (110–180)	147 (107–453)	127 (110–177)	0.277
Creatinin (mg/dL)	66	0.5 (0.4–0.6)	0.5 (0.4–0.6)	0.5 (0.4–0.6)	0.828
Urea (mg/dL)	65	26.7 ± 7.4	27.7 ± 7.2	26.4 ± 7.5	0.527
eGFR (mL/min/1.73 m [2])	66	107 ± 25.4	108 ± 21.0	106 ± 26.9	0.794
Serum albumin (g/L)	66	42 (38–45)	43 (21–47)	42 (39–45)	0.631
Serum protein (g/L)	64	68 (61–73)	69 (44–73)	67 (62–74)	0.374
AST (U/L)	60	32 (27–39)	32 (27–34)	33 (27–42)	0.618
ALT (U/L)	59	24 (19–32)	25 (19–32)	23 (19–32)	0.636
GGT (U/L)	61	43 (31–67)	39 (26–65)	48 (32–67)	0.335
Total bilirubin (mg/dL)	58	0.6 (0.4–0.8)	0.6 (0.2–1.0)	0.6 (0.5–0.8)	0.874
NTproBNP (ng/L)	48	91 (47–224)	122 (42–338)	88 (47–220)	0.709
BNP (ng/L)	12	31 (21–606)	201 (39–778)	22 (19–32)	0.062
Elevated NTproBNP or BNP	60				0.240
Yes		12 (20.3%)	4 (33.3%)	8 (17%)	
No		47 (79.7%)	8 (66.7%)	39 (83%)	
IgG (g/L)	56	8.5 ± 4.0	6.1 ± 3.8	9.0 ± 3.9	**0.038**
TSH (mU/L)	67	3.3 (2.4–4.4)	5.3 (4.9–6.8)	3.1 (2.3–3.6)	**<0.0001**
fT3 (ng/L)	55	3.8 (3.4–4.1)	4.1 (3.6–4.2)	3.8 (3.4–4.1)	0.154
fT4 (ng/L)	67	15.1 (13.0–18.0)	14.1 (11.2–17.9)	15.1 (13.1–18.2)	0.171
fT3/fT4-ratio	55	0.3 (0.2–0.3)	0.3 (0.2–0.3)	0.2 (0.2–0.3)	0.348

Data are presented as n (%) for categorical variables and median [25th – 75th interquartile range] or mean ± standard deviation for continuous variables. ALT = alanine transaminase; AST = aspartate aminotransferase; BNP = brain natriuretic peptide; eGFR = estimated glomerular filtration rate; fT3 = free triiodothyronine; fT4 = free thyroxine; GGT = gamma-glutamyl transferase; IgG = immunoglobulin G; MCV = mean corpuscular volume; NTproBNP = N-terminal pro-B-type natriuretic peptide; TSH = thyroid-stimulating hormone; WBC count = white blood cell count.

**Table 3:** Clinical, hemodynamic and laboratory findings associated with thyroid function tests.

	TSH (mU/L)	fT3 (ng/L)	fT4 (ng/L)	fT3/fT4-ratio
**NYHA class (P-value)**	**0.005**	0.328	0.563	0.130
Class I	3.1 (2.3–3.8)	3.8 (3.5–4.1)	15.2 (13.0–18.1)	0.26 (0.21–0.30)
Class ≥ II	4.1 (3.1–5.5)	3.6 (3.2–4.1)	15.1 (12.8–16.5)	0.24 (0.19–0.26)
**Ventricular dysfunction (P-value)**	0.972	0.801	0.514	0.452
None or mild	3.3 (2.4–4.5)	3.8 (3.5–4.1)	15.1 (12.8–18.0)	0.26 (0.21–0.30)
Moderate or severe	3.3 (2.5–4.4)	3.8 (3.4–4.1)	15.1 (14.0–18.1)	0.25 (0.19–0.29)
**AVV regurgitation (P-value)**	**0.023**	0.107	0.089	**0.009**
No or mild	3.1 (2.3–3.9)	3.8 (3.5–4.1)	14.2 (12.5–17.7)	0.27 (0.22–0.30)
Moderate or severe	4.0 (3.1–5.1)	3.5 (3.2–4.1)	15.5 (14.5–19.6)	0.22 (0.17–0.26)
**Systemic right ventricle (P-value)**	0.100	0.240	**0.005**	**0.007**
Yes	3.6 (2.6–4.7)	3.6 (3.3–4.1)	16.0 (13.8–19.2)	0.22 (0.18–0.28)
No	2.9 (2.3–3.9)	3.9 (3.6–4.1)	13.3 (12.1–16.0)	0.28 (0.25–0.30)
**NTproBNP or BNP (P-value)**	**0.031**	0.371	0.061	0.147
Elevated	3.9 (3.1–5.2)	3.5 (3.3–4.1)	16.0 (15.1–20.4)	0.23 (0.19–0.26)
Normal	3.1 (2.3–3.7)	3.8 (3.5–4.1)	14.3 (13.0–17.7)	0.26 (0.21–0.30)
**PLE diagnosis (P-value)**	**0.002**	0.966	0.260	0.520
Yes	3.9 (3.3–5.2)	3.8 (3.3–4.1)	15.1 (10.9–18.0)	0.25 (0.17–0.29)
No	3.1 (2.3–3.9)	3.8 (3.5–4.1)	15.1 (13.0–18.0)	0.26 (0.20–0.30)
**PLE severity (P-value)**	**0.003**	*	0.780	*
Active disease	4.9 (3.9–5.8)		15.6 (10.4–18.1)	
Remission	3.3 (2.9–4.3)		14.0 (10.8–17.4)	
**Renal function (P-value)**	0.435	0.974	0.230	0.353
eGFR <90 ml/min/1.73 m^2^	3.5 (2.7–4.6)	3.4 (3.1–4.2)	14.6 (12.6–17.1)	0.25 (0.19–0.33)
Normal	3.2 (2.4–4.4)	3.8 (3.5–4.1)	16.0 (13.0–18.2)	0.26 (0.21–0.29)

Data are presented as median [25th – 75th interquartile range]. AVV = atrioventricular valve; BNP = brain natriuretic peptide; eGFR = estimated glomerular filtration rate; fT4 = free thyroxine; fT3 = free triiodothyronine; NTproBNP = N-terminal pro-B-type natriuretic peptide; NYHA = New York Heart Association classification; PLE = protein-losing enteropathy; TSH = thyroid-stimulating hormone; * number of PLE patients with active disease and available serum fT3 levels too low for analysis (n = 4).

The fT3/fT4 ratio was significantly decreased in patients with significant AVV regurgitation (p = 0.009) and in those with a SRV (p = 0.007). Of notice, significant AVV regurgitation was more common in patients with a SRV compared to patients with other SV morphologies (SRV: 15/42 (35.7%) vs non-SRV: 3/25 (12.0%), p = 0.047). Furthermore, patients with a SRV had significantly higher fT4 serum levels (p = 0.005), suggesting that the conversion of T4 to T3 in peripheral tissues may be impaired in these patients.

Significant correlations between thyroid function tests and clinical or laboratory variables are presented in Table 4. A strong inverse relationship was observed between fT3/fT4 ratio and BNP levels (r: −0.599, p = 0.040); of notice, the correlation between fT3/fT4 ratio and NTproBNP levels showed a trend towards statistical significance (r: −0.302, p = 0.052). We also found significant associations between fT3/fT4 ratio and renal function, and serum albumin, protein, and IgG levels. Interestingly, we did not find any association between thyroid function (TSH, fT3, fT4, fT3/fT4 ratio) and surrogate serum markers of systemic inflammation (NLR, PLR) or liver function tests (y-GT, bilirubin, AST, ALT) (all p > 0.05).

**Table 4:** Significant correlations between thyroid function and other variables.

Variable	TSH (mU/L)		fT3 (ng/L)		fT4 (ng/L)		fT3/fT4-ratio	
	*r*	P-value	*r*	P-value	*r*	P-value	*r*	P-value
Oxygen saturation (%)	−0.299	0.015	–	–	–	–	–	–
Weight Z-score	−0.275	0.027	–	–	–	–	–	–
Height z-score	–	–	0.311	0.022	–	–	–	–
BSA	–	–	–	–	−0.293	0.018	–	–
BMI	–	–	–	–	−0.272	0.030	–	–
BMI z-score	–	–	–	–	−0.258	0.040	–	–
Hemoglobin (g/dL)	0.365	0.002	–	–	–	–	–	–
Platelet (x10^9^/L)	–	–	0.380	0.004	–	–	–	–
eGFR (mL/min/1.73 m [2])	–	–	–	–	–	–	0.306	0.023
Serum albumin (g/L)	–	–	–	–	–	–	0.497	<0.001
Serum protein (g/L)	–	–	–	–	−0.330	0.008	0.561	<0.001
NTproBNP (ng/L)	–	–	–	–	0.412	0.004	−0.302	0.052*
BNP (ng/L)	–	–	–	–	–	–	−0.599	0.040
IgG (g/L)	–	–	–	–	−0.269	0.047	0.379	0.008

BMI = body mass index; BNP = brain natriuretic peptide; BSA = body surface area; eGFR = estimated glomerular filtration rate; IgG = immunoglobin G; NTproBNP = N-terminal pro-B-type natriuretic peptide; * = correlation between NTproBNP levels and fT3/fT4-ratio showed a trend towards statistical significance.

We performed a binary logistic regression analysis to identify potential predictors of SHT using a model composed of significant covariates, including NYHA class, history of PLE, systolic BP and weight z-score. In this model, NYHA class ≥ II was an independent predictor of SHT in our Fontan cohort (OR 4.2; 95% CI 1.1–16.1, p = 0.036).

## 4 Discussion

In the present study, SHT was found in 23.9% of our Fontan patients. Patients with SHT had lower systolic BP, a lower body weight z-score, were in a worse NYHA functional class, were more often pacing dependent, and were more likely to have a diagnosis of PLE. Furthermore, a NYHA functional class ≥ II was an independent predictor of SHT in our cohort. Subclinical thyroid dysfunction was also found in patients with significant AVV regurgitation, RV dominance, and in those with elevated NTproBNP or BNP levels, further suggesting an important relationship between the thyroid-axis and hemodynamic derangements following Fontan palliation.

Although previous studies have shown a high prevalence of SHT in children and adults with congenital heart disease, there is only a limited number of studies on thyroid-axis function in Fontan patients [2,[5], [6], [7],[29], [30], [31]]. In addition, most papers only report a small number of Fontan patients, or lack a clear definition of thyroid dysfunction [30,31]. To the best of our knowledge, we present the largest cohort of pediatric Fontan patients in whom thyroid function was investigated. We not only confirm that SHT is common in Fontan patients, but also show that a worse NYHA class was an independent risk factor for SHT. Although the effect of SHT, a clinical state in which the amount of circulating thyroid hormones is not optimal for that patient, on Fontan outcome was not investigated in this paper, it should be mentioned that there is a large body of evidence linking SHT in patients with cardiac disease to accelerated atherosclerosis, adverse outcome and increased mortality [3,4]. However, the associations between SHT and atherosclerosis or clinical outcome have not been found in all studies on this topic, probably due to differences in study design and study population [4]. Therefore, at this point, it is difficult to extrapolate these findings to the Fontan population, and suggesting that subclinical thyroid dysfunction may have a significant clinical impact on Fontan outcome as well. In addition, it is currently not known whether thyroid dysfunction is the cause or effect of hemodynamic derangements in Fontan. Future studies are needed to address these important clinical issues. Furthermore, a recent study showed that patients with cyanotic congenital heart disease and SHT were at risk for the development of overt hypothyroidism [5]. However, there are no studies on the long-term outcome of Fontan patients with thyroid function abnormalities, and whether they are at increased risk for the development of overt hypothyroidism as well.

The mechanism for the association between thyroid function and the hemodynamic findings observed in this study may be explained by the direct effects of thyroid hormones on the cardiovascular system. Normally, T4, and a small proportion of T3, are produced by the thyroid gland under regulation of TSH, with 80% of T3 being derived from deiodination of T4 to T3 in peripheral tissues, such as the kidney, liver, brain and skeletal muscle [32,33]. T3 is the most relevant thyroid hormone in the cardiovascular system, as it is both an important regulator of cardiac-specific genes, and has direct (non-genomic) effects on cardiac ion channels, cell organelles and cytoskeletal components [32,33]. Because cardiomyocytes are not able to convert T4 to T3, even small changes in T3 serum levels may therefore have major impact on cardiovascular function and hemodynamics [32,33]. Indeed, recent studies have shown that lower circulating fT3, a common finding in cardiac disease, causes impaired myocardial contractility, diastolic dysfunction, increased systemic vascular resistance and endothelial dysfunction [3,4,32]. We observed significantly lower fT3 levels in Fontan patients with significant AVV regurgitation and/or a SRV, both of which are well-known risk factors for adverse outcome and development of a failing Fontan circulation [34,35]. AVV regurgitation increases the preload and decreases the cardiac output of the univentricular heart, leading to ventricular dilation, myocardial adverse remodeling, and systolic dysfunction [34]. This may in turn activate the sympathetic nervous system and neurohumoral pathway, contributing to a state of chronic heart failure [34]. Interestingly, we did find a significant relationship between lower T3 serum values and elevation of NTproBNP or BNP levels in our patients, suggesting that an activated neurohumoral system is associated with thyroid dysfunction. Another important observation was that patients with RV dominance not only had lower T3 levels, but were also found to have significantly higher fT4 serum levels. Impaired peripheral conversion of T4 to T3 might explain this finding [33].

PLE may develop in 5–10% of Fontan patients [11,15,21]. The hallmark feature of PLE is excessive enteric protein-loss, resulting in hypoalbuminemia, low oncotic pressure, and peripheral edema or ascites [11,15]. Lymphatic abnormalities in the gut, chronic inflammation, and low cardiac output with impaired mesenteric blood flow may all contribute to the development and progression of PLE. PLE is a heterogeneous systemic disease, associated with high morbidity and poor outcome [11,15]. Therefore, identifying potential modifiable mechanisms of disease in these patients seems essential [21]. This is the first study demonstrating thyroid abnormalities in Fontan patients with PLE. SHT was found in 42.1% of PLE patients. Interestingly, patients with active PLE had significantly higher TSH levels than those who were in remission. Given the fact that the vast majority of thyroid hormone is bound to thyroid hormone-binding globulin, and, to a lesser extent, albumin [33], significant enteric protein-loss in PLE may cause thyroid dysfunction and alterations in thyroid-axis homeostasis. Indeed, strong associations were found between serum protein, serum albumin and IgG levels, and lower circulating fT3 levels. Systemic inflammation and use of certain medications may also contribute to thyroid dysfunction by impairing peripheral conversion of T4 to T3 [33]. A recent study by Manning et al. [36] demonstrated that the Fontan circulation is associated with chronic inflammation, which is exaggerated in Fontan patients with PLE. Although we did not find an association between surrogate markers of inflammation and thyroid function in our study, it should be mentioned that we did not measure pro-inflammatory cytokines such as interleukin-6 (IL-6) or tumor necrosis factor alpha (TNF-α), which may give better insight in the inflammatory status. Based on these data we hypothesize that thyroid dysfunction in PLE is probably secondary to enteric protein-loss, and suggest that other disease mechanisms such as inflammation, may also play a role in its development. Furthermore, systemic disease by itself may also contribute to subclinical thyroid dysfunction [33]. It is also important to note that the effect of thyroid dysfunction on the outcome of Fontan patients with PLE is still unknown.

Renal dysfunction has been demonstrated in 20% of Fontan patients, but its determinants remain not well defined [28,37]. In our study, we found that greater renal dysfunction seems associated with lower fT3 serum levels. Recent studies suggest that thyroid dysfunction may adversely impact renal structure and function, and that kidney disease in itself, may cause thyroid dysfunction through multiple pathways [33]. Taken together, based on recent evidence, data from our present study, and the known direct cardiovascular effects of thyroid hormone, we hypothesize that the thyroid-axis may have an important role in maintaining optimal Fontan hemodynamics and preserving end-organ function in this patient population.

### 4.1 Study limitations

This study has several limitations. First, the sample size of our study was relatively small and some data were lacking. Second, there is a possibility that the patients included in this study created a biased sample, favoring those with failing Fontan hemodynamics and other complications. Third, ventricular dysfunction was not associated with thyroid abnormalities in our study, however, it should be mentioned that ventricular function was assessed only by “eyeballing”. More sophisticated echocardiographic methods such as strain imaging may have given more insight in the relationship between thyroid function and myocardial contractility. Fourth, although we used a recommended cut-off for TSH in our study, it remains unclear what TSH cut-off is appropriate in Fontan patients to define thyroid dysfunction. Despite these limitations, this study reports novel findings and important correlations between thyroid dysfunction and clinical data, particularly in Fontan patients with PLE.

### 4.2 Conclusions

In conclusion, subclinical thyroid dysfunction is common in pediatric Fontan patients and seems associated with unfavorable hemodynamic status and active PLE. We found a complex bidirectional interplay between thyroid function and the hemodynamic status of the Fontan circulation. Even subclinical thyroid dysfunction may directly or indirectly contribute to end-organ damage in this already vulnerable patient population. However, future studies are needed to address the clinical and prognostic implications of (subclinical) thyroid function abnormalities in this population.

## Author contributions

Joszi Sweer: Methodology, Formal analysis, Investigation, Data curation, Writing – original draft, Visualization. Ingo Germund: Conceptualization, Methodology, Validation, Investigation, Resources, Data curation, Writing – Reviewing and Editing, Project administration. Markus Khalil: Investigation, Resources, Data curation, Writing – Reviewing and Editing, Supervision, Project administration, Funding acquisition. Christian Apitz: Investigation, Resources, Data curation, Writing – Reviewing and Editing, Supervision, Project administration. Kim ten Dam: Investigation, Writing – Reviewing and Editing. Stefanie Wendt: Methodology, Writing – Reviewing and Editing. Narayanswami Sreeram: Methodology, Writing – Reviewing and Editing, Supervision. Floris E.A. Udink ten Cate: Conceptualization, Methodology, Validation, Formal analysis, Investigation, Data curation, Writing – original draft, Writing – Reviewing and Editing, Visualization, Supervision, Funding acquisition.

## Sources of funding

Stiftung KinderHerz, Essen, Germany, provided financial support for this study. The funding source did not have any other involvement in this project.

## Declaration of competing interest

The authors declare the following financial interests/personal relationships which may be considered as potential competing interests:

Markus Khalil reports financial support was provided by Stiftung KinderHerz, Essen, Germany.
